# Hydrogel-Based Therapies for Ischemic and Hemorrhagic Stroke: A Comprehensive Review

**DOI:** 10.3390/gels10070476

**Published:** 2024-07-18

**Authors:** Alexandra-Daniela Rotaru-Zăvăleanu, Venera Cristina Dinescu, Madalina Aldea, Andrei Gresita

**Affiliations:** 1 Department of Epidemiology, University of Medicine and Pharmacy of Craiova, 2-4 Petru Rares Str., 200349 Craiova, Romania; alexandra.rotaru@umfcv.ro; 2 Experimental Research Centre for Normal and Pathological Aging, University of Medicine and Pharmacy of Craiova, 200349 Craiova, Romania; agresita@nyit.edu; 3 Department of Health Promotion and Occupational Medicine, University of Medicine and Pharmacy of Craiova, 2–4 Petru Rares Str., 200349 Craiova, Romania; 4 Psychiatry Department, University of Medicine and Pharmacy of Craiova, 200349 Craiova, Romania; 5 Department of Biomedical Sciences, New York Institute of Technology, College of Osteopathic Medicine, Old Westbury, NY 115680, USA

**Keywords:** stroke, hydrogels, biomaterials, neuroprotection, drug delivery, tissue engineering

## Abstract

Stroke remains the second leading cause of death and a major cause of disability worldwide, significantly impacting individuals, families, and healthcare systems. This neurological emergency can be triggered by ischemic events, including small vessel arteriolosclerosis, cardioembolism, and large artery atherothromboembolism, as well as hemorrhagic incidents resulting from macrovascular lesions, venous sinus thrombosis, or vascular malformations, leading to significant neuronal damage. The resultant motor impairment, cognitive dysfunction, and emotional disturbances underscore the urgent need for effective therapeutic interventions. Recent advancements in biomaterials, particularly hydrogels, offer promising new avenues for stroke management. Hydrogels, composed of three-dimensional networks of hydrophilic polymers, are notable for their ability to absorb and retain substantial amounts of water. Commonly used polymers in hydrogel formulations include natural polymers like alginate, chitosan, and collagen, as well as synthetic polymers such as polyethylene glycol (PEG), polyvinyl alcohol (PVA), and polyacrylamide. Their customizable characteristics—such as their porosity, swelling behavior, mechanical strength, and degradation rates—make hydrogels ideal for biomedical applications, including drug delivery, cell delivery, tissue engineering, and the controlled release of therapeutic agents. This review comprehensively explores hydrogel-based approaches to both ischemic and hemorrhagic stroke therapy, elucidating the mechanisms by which hydrogels provide neuroprotection. It covers their application in drug delivery systems, their role in reducing inflammation and secondary injury, and their potential to support neurogenesis and angiogenesis. It also discusses current advancements in hydrogel technology and the significant challenges in translating these innovations from research into clinical practice. Additionally, it emphasizes the limited number of clinical trials utilizing hydrogel therapies for stroke and addresses the associated limitations and constraints, underscoring the need for further research in this field.

## 1. Background

Stroke represents the second leading cause of death and a major cause of disability worldwide, exerting profound impacts on individuals, their caregivers, and the entire healthcare system [1]. This neurological emergency is characterized by the sudden disruption of the blood flow to the brain, due to either a blockage in the cerebral vasculature, caused by small vessel arteriolosclerosis, cardioembolism, or large artery athero-thromboembolism (ischemic stroke), or the rupture of a blood vessel, most frequently due to macrovascular lesions (vascular malformations, aneurysms, cavernomas) or venous sinus thrombosis (hemorrhagic stroke) [2]. Ischemia or hemorrhage can cause extensive neuronal damage, which can lead to a spectrum of debilitating deficits, such as motor impairment (spasticity and weakness) [3], cognitive dysfunction (attention, memory, language, and orientation dysfunction) [4], and even emotional disturbances (post-stroke depression) [5]. Furthermore, cellular balance is severely affected, as glial cells proliferate in response to injury, contributing to a prolonged inflammatory environment that can persist for weeks. This inflammatory response and glial cell proliferation exacerbate the disruption of the neurovascular unit, further complicating the recovery process [6].

In recent years, novel therapeutic approaches such as stem cell therapy [7], pharmaceutical interventions [8], and cellular reprogramming [9] have emerged as viable candidates for post-stroke recovery. Among these, the expanding field of biomaterials has opened up numerous innovative paths for the management and treatment of stroke, with hydrogels demonstrating exceptional potential and emerging as promising solutions for current therapeutic challenges [10]. Hydrogels are complex structures composed of three-dimensional networks of hydrophilic polymers, which are well known for their extraordinary ability to absorb and preserve substantial quantities of water [11]. These polymers have an increased affinity for water molecules, creating a porous structure that enables them to trap and hold water-based solutions within their structure [12]. Hydrogels have the remarkable ability to expand to multiple times their original size while still preserving their structural integrity. This quality renders them indispensable for a wide array of biomedical uses, such as facilitating drug delivery systems, crafting wound dressings, constructing tissue engineering scaffolds, and serving as carriers for the controlled release of therapeutic agents [13]. Moreover, the highly customizable characteristics of hydrogels, including their porosity, swelling behavior, strength, and ability to degrade, can be tailored by selecting specific polymers and employing various crosslinking methods [11]. Notably, these materials can closely mimic the natural extracellular matrix (ECM) by incorporating molecules such as collagen, hyaluronic acid, and fibrin, giving them a wide range of desirable qualities for use in biomedical applications [14]. Importantly, hydrogels are highly biocompatible, making them ideal for targeted treatment and tissue engineering research [15]. Furthermore, this customization and biocompatibility enhance their versatility and practicality in both biomedical research and clinical settings [16]. Given these qualities, researchers and clinicians have taken significant interest in how hydrogels could transform the way we approach stroke treatment.

However, despite their significant potential, biomaterials face several limitations that need to be addressed. Key issues include the potential for immunogenic responses, the difficulty of achieving precise control over drug release rates, and the challenges in ensuring consistent degradation rates in vivo [13]. Moreover, the scalability of hydrogel production for clinical applications remains a substantial challenge, as does the necessity of ensuring their long-term stability and effectiveness within the human body [17]. This review aims to provide a comprehensive overview of hydrogel-based treatments for both ischemic and hemorrhagic stroke. It discusses the complex mechanisms by which hydrogels confer neuroprotection and facilitate drug delivery, while also addressing recent advancements and persistent challenges in translating these innovations from research into clinical practice. Notably, there is a significant imbalance in the volume of research, with the majority of studies focusing on ischemic stroke compared to hemorrhagic stroke. This disparity highlights a critical area that future research needs to address. Furthermore, the relatively low number of clinical trials investigating hydrogel-based therapies underscores the necessity for more extensive clinical validation. By conducting a detailed examination of the relevant literature, this review aims to provide an up-to-date perspective on navigating the evolving field of hydrogel-based stroke therapies. This comprehensive analysis is intended to be a valuable resource for clinicians and researchers.

## 2. Method

**Figure 1:**
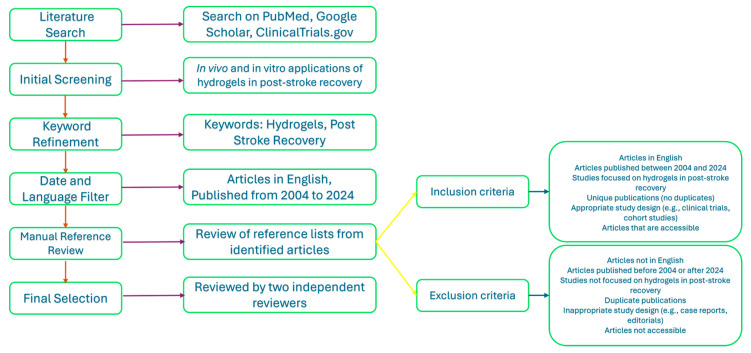
Review process for evaluating the applications of hydrogels in post-stroke recovery.

This review utilized a robust database comprising scientific manuscripts sourced from an exhaustive literature search on platforms like PubMed, Google Scholar, and ClinicalTrials.gov as observed in Figure 1. The emphasis of this review was on both in vivo and in vitro applications of hydrogels in post-stroke recovery. The search was refined using keywords such as “Hydrogels” and “Post Stroke Recovery”. The selection was limited to articles published in English from 2004 to 2024, ensuring contemporary relevance. A manual review of the reference lists from all the identified articles was also conducted to ensure comprehensive coverage of the topic. This narrative review does not follow the Preferred Reporting Items for Systematic Reviews and Meta-Analysis (PRISMA) guidelines but maintains a structured approach to article selection, with each manuscript rigorously evaluated by a panel of two independent reviewers.

## 3. The Pathophysiology of Stroke

Stroke, encompassing both ischemic and hemorrhagic types, represents a complex medical phenomenon that can result in a significant disbalance in the neurovascular unit, leading to impairments in cognitive and motor functions [18]. Ischemic strokes, which account for 80–90% of cases, involve blockage of the blood flow to the brain, leading to tissue death and loss of function in the affected regions [19]. An ischemic event triggers a cascade of molecular processes, including excitotoxicity, oxidative stress, and neuroinflammation [20]. Excitotoxicity, caused by an excess of glutamate release and the resulting activation of glutamate receptors, leads to heightened neuronal activity and an influx of calcium ions, ultimately resulting in cell death [21]. Oxidative stress worsens neuronal damage by encouraging the production of reactive oxygen species (ROS) and triggering lipid peroxidation, protein oxidation, and DNA damage [22]. At the same time, neuroinflammation plays a crucial role in secondary injury, as it involves the activation of microglia, astrocytes, and immune cells that infiltrate the affected area [23]. These inflammatory agents contribute to tissue damage by releasing pro-inflammatory cytokines, chemokines, and cytotoxic substances, which further worsen neurovascular dysfunction and neuronal death [24].

On the other hand, hemorrhagic strokes are characterized by bleeding into the brain, resulting in the extravasation of blood into the cerebral parenchyma or the subarachnoid space. This bleeding increases intracranial pressure and damages the surrounding tissues, often leading to severe neurological deficits [25]. Hemorrhagic strokes are responsible for the disruption of the blood–brain barrier (BBB), the activation of inflammatory pathways, the release of neurotoxic substances from ruptured blood vessels, and the induction of oxidative stress [26]. The disruption of the blood–brain barrier compromises its integrity, allowing harmful substances such as free hemoglobin and iron to infiltrate the brain tissue and exacerbate neuronal injury [27]. Rupture also activates inflammatory pathways, leading to the activation of the immune cells and the release of pro-inflammatory molecules, including cytokines (e.g., interleukin-1β and interleukin-6) and tumor necrosis factor-alpha (TNF-α), which further damage brain tissue [28]. Furthermore, the release of neurotoxic substances from ruptured blood vessels, such as hemoglobin and iron, induces oxidative stress and promotes free radical formation [29]. The presence of blood in the brain tissue results in an imbalance between reactive oxygen species (ROS) production and antioxidant defenses, causing damage to cell membranes, proteins, and DNA, thus contributing to neuronal injury and impaired recovery [30]. This cascade of events directly harms neurons and other brain cells, such as astrocytes and microglia, exacerbating the overall injury and complicating recovery.

**Figure 2:**
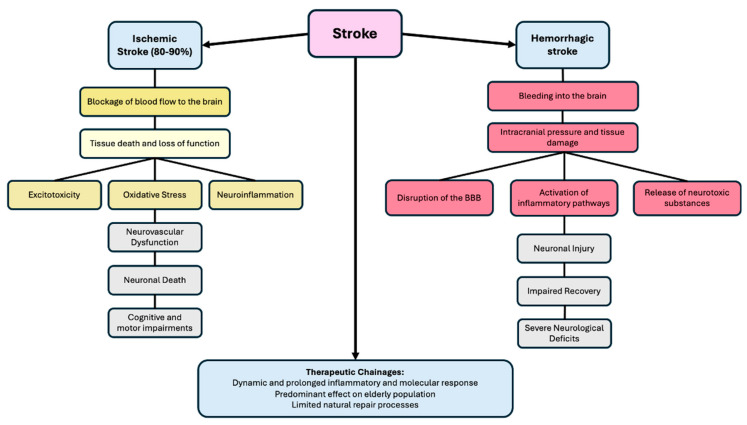
Schematic representation of stroke pathophysiology.

Therapeutic interventions are consequently challenged by rapid and extensive inflammatory and molecular processes that are both highly dynamic and prolonged. Addressing these challenges is particularly difficult given that stroke predominantly affects the elderly population, in whom natural repair processes are further limited (Figure 2) [31].

## 4. Current Treatment Approaches and Emerging Therapies for Stroke

### 4.1. Current Materials Used in Stroke Treatments

In the realm of intravenous and endovascular therapies, the primary material for intravenous thrombolysis is tissue plasminogen activator (tPA), a protein that plays a crucial role in breaking down blood clots [32]. For endovascular thrombectomy, specialized catheters and stents, typically made from biocompatible metals like nitinol and stainless steel, are employed to mechanically remove clots [33]. Surgical interventions in stroke treatment utilize materials such as surgical clips and coils, which are often made from biocompatible metals like platinum, titanium, or stainless steel. These materials are used to clip aneurysms or coil ruptured vessels [34]. Additionally, hemostatic agents, including oxidized cellulose and gelatin-based products, are vital for controlling bleeding during and after surgery [35].

Stem cell therapies and cellular reprogramming involve the use of scaffolds and matrices composed of natural or synthetic polymers such as collagen, fibrin, and hyaluronic acid. These materials provide a supportive environment for cell growth and differentiation. Hydrogels and biodegradable nanoparticles are also used as delivery vehicles to transport stem cells or reprogramming factors directly to the brain tissue [36].

For gene therapy and exosome-based therapies, viral vectors such as adeno-associated viruses (AAVs) and lentiviruses are commonly employed to deliver genetic material into cells [37]. Non-viral vectors, including lipid nanoparticles, are also being explored. Exosome carriers, naturally occurring vesicles composed of lipid bilayers similar to cell membranes, are engineered to carry therapeutic agents [38].

**Figure 3:**
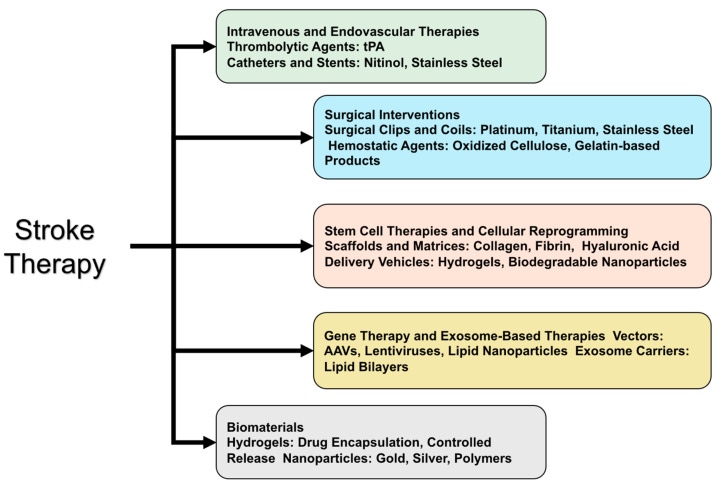
Materials used in stroke treatment.

Biomaterials like hydrogels are utilized for their ability to encapsulate drugs, cells, and growth factors, providing controlled release and protecting therapeutic agents from degradation [39]. Additionally, nanoparticles made from materials such as gold, silver, and various polymers are used to deliver drugs directly to target sites in the brain, enhancing the precision and efficacy of stroke treatments [40] (Figure 3).

### 4.2. Current Treatment Approaches

The primary therapeutic approach to ischemic stroke (IS) involves promptly removing the thrombus to restore blood flow and prevent irreversible damage to the brain cells in the affected and surrounding areas [41]. Currently, the only FDA-approved pharmacological therapy for IS is the intravenous administration of tissue plasminogen activator (tPA) [42]. However, due to its narrow treatment window (<4.5 h) and associated risk of bleeding, only a small percentage of patients (<5%) can benefit from this intervention [43]. Endovascular thrombectomy (ET) is another standard therapy but is similarly constrained by a short therapeutic window (<6 h) and accessibility challenges in some hospitals [44]. Both treatments can lead to cerebrovascular complications and increase the risk of hemorrhagic transformation [45]. Moreover, successful revascularization can result in ischemia/reperfusion injury due to excessive reactive oxygen species production [46]. Overall, the existing clinical approaches to improving the long-term outcomes after IS onset are limited. In the case of hemorrhagic stroke, distinct treatment approaches are required. Surgical interventions, such as aneurysm clipping or coiling and hematoma evacuation, aim to repair the ruptured vessel and relieve intracranial pressure [47]. Pharmacological management includes antihypertensives, anticonvulsants, and agents to reduce cerebral edema [48]. Despite these interventions, the treatment options for hemorrhagic stroke remain limited, and the survival rates are significantly lower compared to those for ischemic stroke. Comprehensive rehabilitation programs are essential to address neurological deficits and optimize recovery in both ischemic and hemorrhagic stroke patients [49].

Despite these established treatment protocols, both ischemic and hemorrhagic strokes continue to pose significant therapeutic challenges due to the limitations and risks associated with current interventions. Consequently, there is a growing need for innovative therapies that can address these gaps and improve patient outcomes. Emerging therapeutic approaches such as stem cell therapies, cellular reprogramming therapy, gene therapy, and the use of exosomes offer promising alternatives [50]. These novel strategies aim to overcome the limitations of traditional treatments by providing more effective and targeted solutions. Preclinical studies have outlined the therapeutic efficacy of diverse stem cell varieties, notably mesenchymal stem cells (MSCs) and neural stem cells (NSCs), in mitigating stroke-induced impairments. These mechanisms include neuroprotection, angiogenesis, and immunomodulation [51]. However, despite promising preclinical data, the translation of stem cell therapies into clinical practice faces serious challenges [52], such as poor cell survival, their limited ability to remain viable over the long term, and the risk of tumor formation, which restrict the effectiveness and safety of stem cell treatments for stroke [53]. Moreover, questions regarding the optimal cell type, dosage, method of delivery, and timing of treatment require more clarification to maximize the potential benefits of stem cell therapies in stroke care [54]. Furthermore, in vivo cellular reprogramming using RNA-based gene manipulation, small-molecule-induced conversion, or transcription factors such as NeuroD1, Ascl1, and Neurogenin2 presents a promising avenue for stroke treatment [55]. This strategy focuses on the direct conversion of resident glial cells, particularly astrocytes and microglia, into functional neurons within the brain’s microenvironment [56]. Given the limitations of existing alternative therapies, there is a growing interest in the development of biomaterial-based interventions [17]. Hydrogels, for instance, offer a promising solution due to their ability to deliver, protect, and enhance existing therapeutic agents. Personalized, biocompatible materials such as hydrogels could represent the future of stroke treatment, providing a versatile and effective platform for improving current and future therapeutic approaches (Table 1) [57].

### 4.3. Other Emerging Therapies

Nanoparticle-Based Therapies: These therapies use nanoparticles to deliver drugs directly to affected brain regions, enhancing drug stability and targeting. However, potential toxicity and regulatory hurdles need to be overcome [58,59]. Gene Editing Technologies (e.g., CRISPR-Cas9): These technologies allow for precise genetic modifications to correct mutations or regulate genes involved in stroke pathology. Challenges include ethical concerns, off-target effects, and the delivery mechanisms [60]. Combination Therapies: Combining stem cells, gene therapy, and pharmacological agents could enhance therapeutic outcomes. However, the complexity of the treatment protocols and the potential for increased side effects must be managed [61]. Bioengineered Scaffolds: These structures mimic the extracellular matrix, promoting tissue regeneration and repair, but their scalability and long-term stability remain challenges [62]. Personalized Medicine: Tailoring treatments based on individual genetic and physiological profiles aims to optimize efficacy and minimize side effects, though this approach involves high costs and complexity [59].

**Table 1:** An overview of current treatments, emerging biomaterials, and the underlying therapeutic mechanisms for ischemic stroke.

Therapeutic Approach	Description	Limitations	Citations
**Ischemic Stroke (IS) Treatment**			
Intravenous administration of tissue plasminogen activator (tPA)	The only FDA-approved pharmacological therapy for IS; used to dissolve the thrombus and restore blood flow.	Narrow treatment window (<4.5 h), risk of bleeding, and only beneficial for <5% of patients.	[63,64]
Endovascular thrombectomy (ET)	Mechanical removal of the thrombus through a catheter.	Short therapeutic window (<6 h), limited accessibility in some hospitals, and risk of cerebrovascular complications and hemorrhagic transformation.	[65,66]
**Hemorrhagic Stroke Treatment**			
Surgical interventions (e.g., aneurysm clipping or coiling, hematoma evacuation)	Aims to repair the ruptured vessel and relieve intracranial pressure.	Limited treatment options and lower survival rates compared to ischemic stroke.	[67]
Pharmacological management	Use of antihypertensives, anticonvulsants, and agents to reduce cerebral edema.	Limited effectiveness in improving survival rates.	[68]
**Emerging Therapies**			
Stem cell therapies	Utilizes various stem cell types (e.g., MSCs, NSCs) for neuroprotection, angiogenesis, and immunomodulation.	Challenges include poor cell survival, limited long-term viability, risk of tumor formation, and unclear optimal protocols.	[69,70,71,72]
Cellular reprogramming	Direct conversion of resident glial cells into functional neurons using RNA-based gene manipulation, small molecules, or transcription factors (e.g., NeuroD1, Ascl1, Neurogenin2).	Requires more research on the delivery methods and the functional integration of reprogrammed cells.	[73,74]
Gene therapy and exosome-based therapies	Emerging strategies to enhance stroke recovery through targeted genetic modifications or the use of exosomes to deliver therapeutic agents.	High complexity, regulatory challenges, and need for extensive preclinical and clinical validation.	[75]
**Biomaterial-Based Interventions**			
Hydrogels	Biocompatible, customizable materials that can deliver, protect, and enhance therapeutic agents, potentially improving drug delivery, neuroprotection, and tissue regeneration.	Potential for immune response, difficulty in achieving precise drug release and degradation rates, scalability issues, and long-term stability concerns.	[76,77,78]
**Other Therapies**			
Nanoparticle-based therapies	Using nanoparticles to deliver drugs directly to the affected brain regions, enhancing drug stability and targeting.	Potential toxicity, challenges in targeting specific brain regions, and regulatory hurdles.	[58,79]
Gene editing technologies (e.g., CRISPR-Cas9)	Precise genetic modifications to correct mutations or upregulate/downregulate specific genes involved in stroke pathology.	Ethical concerns, off-target effects, and delivery challenges.	[59,60]
Combination therapies	Combining multiple treatment modalities such as stem cells, gene therapy, and pharmacological agents to enhance therapeutic outcomes.	Complexity in treatment protocols, potential for increased side effects, and higher cost.	[61]
**Innovative Approaches**			
Bioengineered scaffolds	Creating supportive structures that mimic the extracellular matrix, promoting tissue regeneration and repair.	Risk of immune response and challenges in scalability challenges and ensuring long-term stability.	[62]
Personalized medicine	Tailoring treatments based on individual genetic and physiological profiles to optimize efficacy and minimize side effects.	High cost, complexity of genetic profiling, and need for extensive patient data.	[60]

## 5. Hydrogels in Biomedicine

### 5.1. The Classification, Properties, and Composition of Hydrogels

Hydrogels are highly versatile materials that have garnered significant interest in various fields, including biomedicine, agriculture, and environmental science (40). These materials are composed of crosslinked polymer chains forming three-dimensional (3D) network structures, capable of absorbing and retaining large amounts of water. This high water content closely mimics the extracellular matrix (ECM) in biological tissues, endowing hydrogels with exceptional biocompatibility and facilitating smooth integration with host tissues with minimal adverse reactions upon implantation [80]. Furthermore, the mechanical properties of hydrogels can be precisely tuned to match specific tissues, making them suitable for various applications, ranging from soft tissue engineering to load-bearing implants [81]. Hydrogels possess a wide array of characteristics and applications, leading to their classification through various criteria. One common approach is based on their composition, distinguishing between natural hydrogels, derived from polymers like collagen or hyaluronic acid, and synthetic hydrogels, which are synthesized from artificial polymers such as polyethylene glycol (PEG) or polyvinyl alcohol (PVA) [82]. Another classification criterion considers the charge of hydrogels, categorizing them as cationic, anionic, or nonionic depending on their electrical properties [83]. Additionally, hydrogels can be grouped based on their crosslinking mechanisms, with physical hydrogels relying on interactions like hydrogen bonding and chemical hydrogels formed through covalent bonds via chemical reactions [84]. The origin of hydrogels is also used for classification, distinguishing between those derived from natural sources like animal or plant extracts and those entirely synthesized in a laboratory setting [85]. Hydrogels’ responsiveness to external stimuli, such as temperature, pH, light, or electrical signals, serves as another basis for classification. For instance, thermoresponsive hydrogels can change their properties in response to temperature variations, and pH-responsive hydrogels can alter their behavior based on the surrounding pH levels. Other stimuli responses include light-responsive hydrogels that react to specific wavelengths of light and electrically responsive hydrogels that respond to electrical signals [86].

In addition to their responsive nature, hydrogels are designed to degrade safely within the body post-therapy, eliminating the need for surgical removal and offering a significant advantage over non-degradable systems [17]. Biodegradable hydrogels, composed of various natural and synthetic polymers, each provide unique benefits for biomedical applications. Natural polymers like alginate, collagen, hyaluronic acid, chitosan, and gelatin are favored for their biocompatibility and similarity to the natural extracellular matrix (ECM) [87]. For instance, alginate, derived from brown seaweed, forms hydrogels with calcium ions and is used for cell delivery, tissue repair, and targeted drug release due to its mild gelation and biocompatibility [88]. Collagen, the most abundant protein in the human body, supports cell adhesion, neurogenesis, and angiogenesis, making it ideal for tissue repair [89]. Hyaluronic acid promotes cell proliferation and migration, mimics the ECM, and effectively delivers neuroprotective agents [90]. Chitosan, with its antimicrobial properties, reduces oxidative stress and supports neuroprotection and tissue repair [91]. Gelatin, derived from collagen, delivers anti-inflammatory agents and supports cell proliferation and neuroprotection [92]. Furthermore, synthetic polymers like PLGA (poly(lactic-co-glycolic acid)), PEG (polyethylene glycol), and PVA (polyvinyl alcohol) offer precise control over the mechanical properties and degradation rates. PLGA is known for its controlled release of anti-inflammatory and neuroprotective agents, supporting neural regeneration. PEG is used for sustained therapeutic agent release, enhancing neuroprotection and tissue repair. PVA, known for its excellent film-forming, emulsifying, and adhesive properties, is utilized in various biomedical applications, including drug delivery systems. These diverse hydrogel compositions provide a foundation for their broad applicability in biomedical fields, particularly in drug delivery and tissue engineering [93] (Table 2). These hydrogels break down into non-toxic by-products that the body can easily eliminate, making them ideal candidates for a wide range of medical applications [94]. The continuous development of hydrogel materials promises to enhance their therapeutic potential and broaden their applicability in the treatment of various medical conditions, including ischemic stroke.

### 5.2. Hydrogel Crosslinking

For effective biomedical use, materials must be biocompatible, biodegradable, and non-toxic. Crosslinking is a critical process in the design and functionality of hydrogels, significantly impacting their mechanical strength, degradation properties, and responsiveness to stimuli [105]. Advanced crosslinking techniques are essential for designing hydrogels with specific mechanical and degradation properties suitable for targeted applications [106]. Various crosslinking methods, including chemical crosslinking with agents like glutaraldehyde or EDC/NHS, physical crosslinking through freeze–thaw cycles or ionic interactions, enzymatic crosslinking using enzymes like transglutaminase, and photocrosslinking using light-sensitive materials, offer diverse options for enhancing the structural integrity and functional properties of hydrogels [107]. Crosslinking not only enhances the structural integrity and functional properties of hydrogels but also allows for the customization of the hydrogel’s behavior to meet specific biomedical needs. For instance, dual-crosslinked hydrogels, which combine both physical and chemical crosslinking methods, offer improved mechanical strength and resilience, making them suitable for load-bearing applications [84]. Additionally, dynamic covalent crosslinking, which involves reversible covalent bonds, enables the creation of self-healing hydrogels that can repair themselves after damage, thereby extending their lifespan and functionality in vivo [108]. Researchers are also exploring bio-orthogonal click chemistry for crosslinking, a method that allows for precise and biocompatible hydrogel formation under physiological conditions without interfering with biological processes. This approach facilitates the incorporation of bioactive molecules and cells into the hydrogel matrix, enhancing their therapeutic potential [109]. Physical hydrogels rely on non-covalent interactions such as hydrogen bonding, ionic interactions, or hydrophobic interactions, while chemical hydrogels are formed through covalent bonds via chemical reactions (51). Internal-stimuli-responsive hydrogels, which are triggered by factors like the pH and temperature naturally present in the body, show great promise for precise drug delivery. These internally triggered hydrogels, particularly when designed with smaller sizes, react more quickly, thereby enhancing drug efficacy [110]. pH-sensitive swelling, achieved through specific polymer modifications, enables targeted drug release in acidic environments, such as those found in certain diseased tissues [94]. To maintain mechanical strength while ensuring a rapid response, researchers are developing novel polymer synthesis methods and exploring the use of non-toxic crosslinkers [111].

Photocrosslinking is a technique used to form and stabilize hydrogels with precise control over their properties. This method involves using light to initiate polymerization, crosslinking polymer chains within the hydrogel. Photocrosslinking offers spatial and temporal control over gelation, beneficial for creating complex structures. It employs photoinitiators like Irgacure 2959, eosin Y, and lithium phenyl-2,4,6-trimethylbenzoylphosphinate (LAP) that generate reactive species upon exposure to specific wavelengths of light, initiating crosslinking. This technique allows for customization of hydrogel properties such as their stiffness, degradation rate, and functionalization with bioactive molecules. Consequently, photocrosslinked hydrogels are extensively used in tissue engineering, drug delivery systems, and wound healing applications, where precise structural and functional requirements are essential [94].

Crosslinking by radical polymerization is a widely used technique to create hydrogels with tailored mechanical and chemical properties. The process involves three key steps: initiation, propagation, and termination [112]. Thermal initiation begins with the generation of free radicals through the thermal decomposition of initiators like azobisisobutyronitrile (AIBN) or benzoyl peroxide. These radicals propagate by rapidly adding monomer units, such as acrylates or methacrylates, extending the polymer chain [113]. Redox initiation involves redox reactions, utilizing reducing agents like ferrous ions and oxidizing agents like hydrogen peroxide to generate radicals for polymerization at lower temperatures, which is beneficial for sensitive biological applications. Controlled radical polymerization techniques offer precise control over the polymerization process. For example, Atom Transfer Radical Polymerization (ATRP) uses transition metal catalysts to manage polymerization, ensuring specific molecular weights and polymer architectures [114]. Reversible Addition–Fragmentation Chain Transfer (RAFT) polymerization employs chain transfer agents, enabling the creation of complex structures with precise properties [115]. Nitroxide-Mediated Polymerization (NMP) utilizes stable nitroxide radicals to regulate the growth of polymer chains, providing excellent control over the polymerization kinetics and molecular weight distribution [116]. Crosslinking occurs when multifunctional monomers form bridges between polymer chains, transforming the solution into a gel. Factors like the monomer-to-crosslinker ratio, initiator concentration, and polymerization conditions (temperature, solvent, pH) significantly influence the hydrogel’s properties [83]. Hydrogels produced this way are used, in general, as drug delivery systems. The method’s advantages include its simplicity and versatility in the monomer selection, though challenges remain in controlling the uniformity and distribution of the crosslinks. Understanding these variables allows for precise tuning of the hydrogel’s characteristics to meet specific application needs [117,118].

In crosslinking through the chemical reaction of complementary groups, water-soluble polymers are soluble due to functional groups like OH, COOH, and NH2, which can be used to form hydrogels. Crosslinking occurs through the covalent bonds between these polymer chains. This is achieved by reacting functional groups with complementary reactivity, such as amine with carboxylic acid and isocyanate with OH or NH2, or through Schiff base formation. These chemical reactions create stable, covalently bonded networks within the hydrogel structure [119].

Crosslinking using high-energy irradiation, particularly gamma and electron beams, can polymerize unsaturated compounds, converting water-soluble polymers with vinyl groups into hydrogels [120]. This process also works with mixtures of monofunctional acrylates and suitable crosslinkers. Additionally, high-energy irradiation can crosslink water-soluble polymers without vinyl groups [121]. During irradiation, radicals form on the polymer chains, and the radiolysis of water produces hydroxyl radicals that attack the polymer chains, forming macroradicals. These recombine, creating covalent bonds and crosslinked structures. This irradiation is typically performed in an inert atmosphere to prevent oxygen interference. Examples include poly(vinyl alcohol), poly(ethylene glycol), and poly(acrylic acid). The resulting hydrogels’ properties, such as their swelling and permeability, depend on the polymer concentration and radiation dose. For instance, thermosensitive hydrogels and polymer microparticles can be prepared by irradiation. The advantage of this method is the ability to form hydrogels in water under mild conditions without toxic crosslinking agents. However, biologically active materials must be loaded post-preparation due to potential damage from irradiation-induced radicals. Additionally, the crosslinks in some polymers, such as PEG or PVA, are non-biodegradable [122].

**Figure 4:**
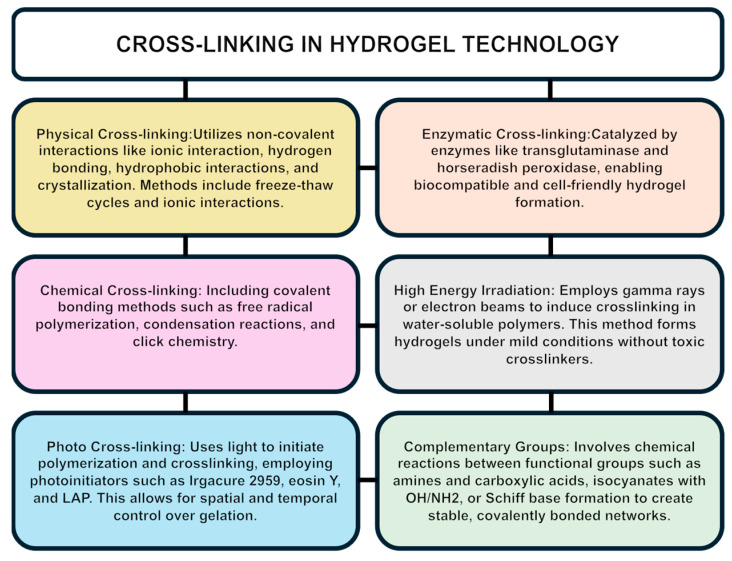
Crosslinking in hydrogel technology.

The diverse crosslinking methods for hydrogels, including chemical, physical, enzymatic, and photocrosslinking, hold potential benefits for stroke therapy [123]. Chemical crosslinking could provide stable and robust hydrogels for sustained drug release, enhancing neuroprotection in ischemic stroke. Physical crosslinking using non-covalent interactions may create hydrogels that respond to internal stimuli like pH changes, enabling targeted drug delivery in the acidic environments of injured brain tissue. Enzymatic crosslinking offers the possibility of biocompatible and cell-friendly hydrogels that promote tissue regeneration. Photocrosslinking allows for precise control over the hydrogel properties, potentially enabling the creation of complex structures for tailored therapeutic applications [107,124]. These crosslinking techniques collectively offer promising avenues for developing hydrogels that enhance drug delivery, support tissue repair, and improve the outcomes in both ischemic and hemorrhagic stroke therapy [107]. Figure 4 depicts a graphic representation of the previously described crosslinking methods.

## 6. Hydrogel Applications in Biomedical Research and Stroke Therapy

In biomedical research and clinical practice, hydrogels are widely utilized for various applications due to their unique properties and versatility [125]. One of the most significant applications of hydrogels is in drug delivery. Their natural porosity and ability to encapsulate therapeutic agents make them ideal for controlled and targeted drug release [126]. By adjusting factors such as the polymer composition, crosslinking density, and degradation rates, researchers can precisely control the drug release profiles from hydrogels, enhancing their therapeutic effectiveness and reducing systemic side effects [11]. Hydrogels offer a versatile platform for delivering a wide range of therapeutics, including small molecules, proteins, nucleic acids, and even cells [127]. Notably, hydrogels have been shown to transport and release therapeutic agents in a controlled manner [128]. In tissue engineering, hydrogels provide scaffolding that supports cell growth and tissue regeneration [129]. Recent studies, such as work by Wand et al., have shown that treating fractures with siRNA/NPs delivered from hydrogels not only increases bone formation but also enhances its biomechanical strength [130]. In oncology, Campea’s research has highlighted the potential of nanogel-based nanoassemblies to enhance the effectiveness and safety of nanoparticle-based drug delivery systems for cancer treatment [131]. Additionally, Yin et al. synthesized phosphate (Pi) and polyphosphate (PPi) crosslinked poly(ethylene glycol) (PEG) hydrogel nanoparticles (NP-Pi and NP-PPi) and demonstrated that NP-PPi effectively suppressed the virulence factors of P. aeruginosa in vitro, suggesting their potential to target pathogenic phenotypes in the gastrointestinal tract [132]. Licht et al. showed that synthetic 3D PEG–Anisogel tailored with fibronectin fragments could lead to linear neurite extension, a significant step toward its clinical translation and the potential treatment of acute spinal cord injuries [133].

In stroke treatment, hydrogel-based approaches to tissue engineering are particularly promising [134]. Scientists can design hydrogels to replicate the biochemical and mechanical signals found in physiological tissue environments, thereby enhancing cell adhesion, proliferation, and differentiation [15]. By incorporating bioactive molecules like growth factors, cytokines, and extracellular matrix proteins, hydrogel scaffolds can improve tissue regeneration and restructuring [135]. Hydrogels can effectively capture and release bioactive molecules in a controlled manner, allowing them to influence the surrounding microenvironment. This modulation can help reduce secondary injury processes and support neuronal survival [74]. For example, hydrogel-based systems can be customized to administer neurotrophic factors such as brain-derived neurotrophic factor (BDNF), antioxidants like vitamin E, or anti-inflammatory agents such as dexamethasone directly to the injury site. This targeted delivery helps reduce oxidative stress, inflammation, and excitotoxicity, all of which contribute to neuronal damage following a stroke [57]. Moreover, hydrogels can act as carriers for cell transplantation, allowing therapeutic cells such as neural progenitor cells or mesenchymal stem cells to be delivered to damaged brain tissue [136]. Transplanted cells enclosed in hydrogels can provide neuroprotective benefits by releasing trophic factors, modulating the immune response, boosting natural repair processes, and aiding in functional recovery after a stroke [137]. However, despite their potential, hydrogels have some limitations. Concerns such as the potential for an immune response [138], the difficulty of achieving precise control over the drug release rates, and challenges in ensuring consistent degradation rates in vivo need to be addressed [139]. Additionally, the scalability of hydrogel production for clinical applications remains a challenge, as does ensuring their long-term stability and effectiveness within the human body [13].

### 6.1. Hydrogels Used in Ischemic Stroke Treatment

#### 6.1.1. Hydrogels as Drug Delivery Systems

Hydrogels offer an adaptable structure for delivering therapeutic agents in a controlled manner, making them highly suitable for biomedical applications [16]. By precisely engineering their composition and structure, hydrogels can be used to release drugs gradually over an extended period, ensuring continuous treatment [11]. This controlled release mechanism is particularly advantageous for administering thrombolytic agents such as tissue plasminogen activator (tPA) or urokinase, which can dissolve the blood clots that obstruct the blood flow in the brain during an ischemic stroke [140]. Additionally, hydrogels can be loaded with anti-inflammatory drugs like dexamethasone or neuroprotective drugs like memantine or erythropoietin to modulate the inflammatory response and minimize neuronal damage following an ischemic stroke [10]. When applied directly to the affected area of the brain, hydrogels optimize the delivery of high concentrations of drugs precisely where they are needed most [141]. This localized delivery approach minimizes the systemic side effects that can occur with oral or intravenous drug administration [142]. By targeting drug delivery to the site of injury, hydrogels enhance the effectiveness of the treatment while reducing the risk of adverse reactions elsewhere in the body [143]. Recent studies, such as those by Jin et al., have demonstrated the potential of hydrogels in stroke therapy. Their research on the delivery of human recombinant osteopontin via biodegradable gelatin microspheres highlights a promising strategy for enhancing neuroprotection in the post-ischemic brain. Gelatin microspheres (GMSs) are popular drug carriers due to their excellent biocompatibility and safe degradation products. The drug release profile can be easily customized by adjusting the crosslinking density and size of the GMSs. In one study, GMSs that were 25 μm in diameter and crosslinked with 0.03125% glutaraldehyde were used to achieve both a rapid initial release and sustained release over time. The release of osteopontin from these microspheres significantly augmented its neuroprotective efficacy [144]. This approach holds immense potential, particularly when considering ongoing research into genetically engineered peptides, cells, and plasmids. Additionally, combining gelatin microsphere delivery with other therapeutic modalities, either simultaneously or in a spatiotemporally controlled manner, is anticipated to further amplify their therapeutic benefits. Hydrogels can also serve as an effective delivery system for growth factors and drugs in nerve repair and regeneration after a stroke. For example, they can be used to deliver growth factors such as brain-derived neurotrophic factor, nerve growth factor (NGF), and glial cell line-derived neurotrophic factor (GDNF) [145]. The targeted application of growth factors may provide an extended therapeutic window to restore tissue damaged by stroke [146]. Moreover, hydrogels can deliver drugs like fibroblast growth factor (FGF) and erythropoietin, which support neurogenesis and neuroprotection. For example, HAMC hydrogel (hyaluronan/methyl cellulose) can deliver erythropoietin (EPO), which stimulates the migration of neural stem cells and mature neuroblasts while reducing apoptosis at the injury site, as demonstrated by Wang et al. HA and MC were dissolved in dH2O at 4 °C overnight and then sterile-filtered and lyophilized. The sterile powders were stored at 4 °C until use. HAMC was prepared with 1.1% HA and 2.2% MC in sterile aCSF and mixed using a SpeedMixer. Before injection, 100 μL of sterile EPO solution (10,000 U/mL) was added to 900 μL of HAMC, resulting in a final concentration of 1% HA and 2% MC. The mixture was then injected into the stroke area to ensure the gel made direct contact with the brain’s cortical surface [147]. Similarly, gelatin hydrogel microspheres (biodegradable gelatin hydrogels that were made by crosslinking acidic gelatin (with an isoelectric point of 5.0) and basic gelatin (with an isoelectric point of 9.0) using glutaraldehyde) were used to deliver insulin-like growth factor-1 and hepatocyte growth factor, increasing the number of new neurons in the subventricular region and those migrating to the damaged striatum, according to Nakaguchi et al. [148]. Injectable hydrogels designed for the slow release of growth factors and drugs can be tailored to meet the specific time and speed requirements of different bioactive substances. This allows for a more controlled slow-release effect, enhancing the therapeutic outcomes [149,150]. Hydrogels offer a sophisticated and effective means to administer medications that alleviate cerebral edema, a common and dangerous complication following ischemic stroke [151]. By reducing swelling, the intracranial pressure is minimized, thereby preventing additional neuronal damage and improving patient outcomes [152]. Interestingly, hydrogels can be engineered to release therapeutic agents that specifically address the underlying causes of brain edema. For instance, they can deliver anti-inflammatory drugs that mitigate inflammation and subsequent swelling. The controlled and sustained release of these drugs from hydrogels ensures a consistent therapeutic effect, which is crucial for alleviating the acute inflammatory response that exacerbates brain swelling [153]. Furthermore, hydrogels can be designed to contain osmotic agents such as mannitol and glycerol, which function by drawing excess fluid away from the brain tissue, thus reducing swelling [127]. This osmotic effect is particularly beneficial in managing intracranial pressure and preventing further neuronal injury. In addition to anti-inflammatory and osmotic agents, hydrogels can be loaded with antioxidants that counteract oxidative stress, another major contributor to cerebral edema. For example, hydrogels incorporating agents like vitamin E or N-acetylcysteine can scavenge free radicals, thereby reducing oxidative damage and subsequent swelling. The versatility and precision of hydrogel-based delivery systems make them a promising tool for future research.

#### 6.1.2. Hydrogels for Neuroprotection and Tissue Repair

Engineered scaffolds create a biocompatible three-dimensional microenvironment that supports the growth and survival of the therapeutic cells seeded onto them. This kind of environment is crucial for assessing the potency and safety of various stem cells (SCs) in stroke treatment. The most commonly used SCs in bioprinting applications include neural stem cells (NSCs), mesenchymal stem cells (MSCs), hematopoietic stem cells (HSCs), induced pluripotent stem cells (iPSCs), and embryonic stem cells (ESCs) [154]. NSCs, known for their neuroprotective properties, have shown significant potential in stroke treatment [155]. For instance, an electrically conductive three-dimensional scaffold was developed as a novel NSC delivery system, which improved neurological recovery post-stroke [156]. Current applications of NSCs to engineered scaffolds, when transplanted into adult rat spinal cords, have shown promising effects such as reducing necrosis in the brain tissue and preventing inflammation and glial scar formation [157]. MSCs, which can replicate the biological three-dimensional network of cells and the extracellular matrix, create an “in vivo-like” microenvironment that is better preserved [158,159]. Recent studies have demonstrated that MSCs can be assembled into spheroid-shaped structures using 3D engineering techniques, enhancing their differentiation into neuronal-like phenotypes [160]. Enhancing the dynamic culture parameters through bioprinting could further improve the construction of 3D SC-seeded scaffolds, making them more efficient [161,162]. The advancement of engineered scaffolds in combination with stem cell therapies holds great promise for improving stroke recovery outcomes. By providing a supportive and conducive environment for stem cell growth and differentiation, these scaffolds not only enhance the therapeutic potential of SCs but also offer a robust platform for further research and development in regenerative medicine [36].

In addition to this, by incorporating growth factors such as brain-derived neurotrophic factor (BDNF) and vascular endothelial growth factor (VEGF), hydrogels can significantly enhance neuron survival, neurogenesis, and angiogenesis [163]. BDNF supports the growth and differentiation of the neurons by activating the TrkB receptor, fostering new neuronal connections crucial for recovery after a stroke [164]. A study by Obermeyer demonstrated that using an encapsulation-free method, BDNF was dispersed in a hydrogel made of hyaluronan and methyl cellulose (HAMC), along with poly(lactic-co-glycolic acid) (PLGA) nanoparticles. This composite was then applied epi-cortically, directly above the stroke lesion, in a rat model of stroke, allowing BDNF to diffuse into the brain, leading to enhanced behavioral recovery and increased synaptic plasticity in the contralesional hemisphere [165]. VEGF promotes the formation of new blood vessels, restoring blood supply to damaged brain areas, which improves oxygen and nutrient delivery [166]. VEGF gels have been shown to significantly reduce lesion volume. In contrast, neither blank gels nor bolus VEGF injections provided any neurological or histological benefits. These findings indicate that delivering vascular endothelial growth factor (VEGF) from a hydrogel directly to the brain can offer substantial functional and structural protection against ischemic damage, as demonstrated in a rat stroke model by Emerich et al. Partially oxidized low-molecular-weight (50 kDa) and high-molecular-weight (MW) (250 kDa) ultrapure MVG alginates were reconstituted in EBM-2 to create a 2% *w*/*v* solution, with a ratio of 75% LMW to 25% HMW used in all the experiments. Before gelation, the alginate solutions were mixed with lyophilized recombinant human VEGF165 protein. This process produced an alginate scaffold that delivered 1 μg of VEGF when injected into the striatum at a volume of 5 μL. This demonstrated that intracerebral implantation of this scaffold provided therapeutic sustained delivery of VEGF in a rodent model of stroke [167]. Similarly, Gu et al. showed that hydrogels support tissue regeneration by promoting cerebral angiogenesis and enhancing neurological function through providing a scaffold for cell growth and organization, which aids in the repair of damaged tissues. A HAD (adhesive hydrogel) precursor was created by grafting DA (3,4-dihydroxyphenylalanine ) and AEMA (2-aminoethyl methacrylate) onto a hyaluronic acid chain, serving as wet adhesive and photocurable groups. Synthesis involved dissolving HA (hyaluronic acid) and adding EDC (3-ethylcarbodiimide hydrochloride), NHS (N-Hydroxy succinimide), AEMA, and dopamine, followed by dialysis and lyophilization [168]. Hydrogels can also release peptides or proteins that inhibit apoptotic pathways, thereby protecting neurons from ischemic damage. Apoptosis is a major cause of neuronal death following a stroke, but by incorporating molecules that block this process, such as caspase inhibitors or anti-apoptotic proteins like Bcl-2, hydrogels can help preserve the neurons. These molecules interfere with apoptotic signaling pathways, preventing cell death and promoting recovery [57]. The controlled release of these bioactive molecules ensures a sustained therapeutic effect, crucial for long-term recovery. Furthermore, hydrogels can be engineered to degrade at specific rates, providing a continuous supply of growth factors and anti-apoptotic agents over time. This feature is particularly beneficial during the chronic phase of stroke recovery, where ongoing support for neurogenesis and angiogenesis is necessary [11]. The ability to tailor the degradation rate of hydrogels enhances their effectiveness in providing prolonged therapeutic benefits, making them ideal for supporting long-term recovery processes.

#### 6.1.3. Hydrogels for Reducing Secondary Injury

One of the critical issues following a stroke is excitotoxicity, which is caused by the excessive release of glutamate, an excitatory neurotransmitter [169]. This glutamate excess overstimulates the NMDA receptors on the neurons, leading to calcium overload and subsequent neuronal death [21]. Hydrogels can be engineered to release NMDA receptor antagonists, which block these receptors and prevent the cascade of excitotoxic damage, thereby protecting the neurons from hyperactivity and death [170]. In addition to excitotoxicity, oxidative stress is a major contributor to cellular damage during ischemic injury [171]. The lack of blood flow leads to an overproduction of reactive oxygen species (ROS), highly reactive molecules that can damage cellular components like DNA, proteins, and lipids [172]. Hydrogels can be loaded with antioxidants, such as superoxide dismutase (SOD) or catalase, which neutralize ROS and reduce oxidative stress [173]. By scavenging these harmful molecules, antioxidants help to protect cells from damage and promote recovery [174]. For example, thymoquinone, known for its antioxidant properties and ability to reduce ROS levels, is quickly eliminated from the plasma when administered systemically. However, using PLGA and chitosan to sustain the delivery of thymoquinone via the intranasal route resulted in improved post-stroke functional outcomes according to Xian et al. [175].

Inflammation is another significant factor that exacerbates injury after a stroke [176]. Activated microglia and astrocytes release pro-inflammatory cytokines and chemokines, which can lead to further neuronal damage and inhibit repair processes [177]. Hydrogels can be designed to deliver anti-inflammatory agents, such as dexamethasone or interleukin-10 (IL-10), directly to the affected area [178]. These agents suppress the activation of the microglia and astrocytes, thereby reducing the inflammatory response and limiting additional damage [179]. Edaravone, an antioxidant and anti-inflammatory drug, is the only neuroprotective compound approved for clinical use in stroke patients in Japan. Recently, Tamer et al. showed the anti-inflammatory benefits of releasing edaravone from composites of hyaluronan and chitosan using both in vitro and in vivo studies. In this study, they developed a new lesion dressing membrane using chitosan, hyaluronic acid, and edaravone, which significantly improved the healing process in rats. Adding edaravone to the chitosan–hyaluronic acid membranes made them less hydrophilic, as shown by their lower water uptake and higher water contact angle [180]. By addressing excitotoxicity, oxidative stress, and inflammation, hydrogels provide a multi-faceted therapeutic approach to improving the outcomes following a stroke. Their ability to deliver targeted treatments directly to the site of injury enhances their effectiveness and offers a promising strategy for neuroprotection and recovery [10].

#### 6.1.4. Hydrogels’ Involvement in Blood–Brain Barrier Protection

Hydrogels, typically composed of natural or synthetic polymers, can be engineered to deliver therapeutic agents that reinforce or repair the blood–brain barrier, which is crucial in protecting the brain tissue from harmful compounds and preventing further damage during ischemic stroke [181]. The BBB is a selective barrier that regulates the passage of substances between the bloodstream and the brain, and its integrity is often compromised during a stroke [27]. Hydrogels can be loaded with drugs such as growth factors, matrix metalloproteinase inhibitors, or tight junction proteins that help restore the structure and function of the BBB [182,183,184]. These hydrogels often involve chemical crosslinking, where covalent bonds are formed between polymer chains, or physical crosslinking, which relies on non-covalent interactions like hydrogen bonding, ionic interactions, or hydrophobic interactions [11].

From a cellular and molecular perspective, these agents work by promoting the expression of proteins that strengthen the tight junctions between endothelial cells, reducing the BBB’s permeability [185]. Growth factors like VEGF can stimulate angiogenesis and the repair of damaged blood vessels, enhancing the overall stability of the BBB, as shown in murine model studies [186]. Additionally, matrix metalloproteinase inhibitors can prevent the degradation of extracellular matrix components, further supporting the BBB’s structural integrity [187]. Hydrogels can also exhibit responsive characteristics, such as being pH-sensitive, temperature-sensitive, or enzyme-sensitive, allowing for targeted and controlled release of therapeutic agents in response to specific stimuli [188].

Just as the native extracellular matrix (ECM) acts as a scaffold for cells in the brain, hydrogel matrices mimic this function in three-dimensional in vitro models. They provide a platform for cell adherence and migration and induce mechanical cues that promote cellular health. The combination of induced pluripotent stem cell (iPSC) technology with hydrogel scaffolds, which emulate the properties of the native ECM, along with advancements like 3D bioprinting, microfluidic devices, and organoid development, has enabled the creation of various models of the neurovascular unit. These models are instrumental for reliably assessing BBB functionality, disease modeling, and drug development [189].

#### 6.1.5. Regulatory Aspects of Hydrogels in the Management of Ischemic Stroke (IS)

Hydrogels present a promising avenue for the treatment of ischemic stroke (IS), but a rigorous regulatory landscape must be navigated to ensure their safety, efficacy, and quality. Before any clinical trials can begin, comprehensive preclinical studies are necessary. These studies involve assessing their biocompatibility, toxicity, and initial efficacy in animal models, with the data thoroughly reviewed by regulatory bodies such as the FDA in the USA and the EMA in Europe [190].

Clinical trials progress through multiple phases, each requiring regulatory approval, and have been so far restricted to preclinical studies. Phase I trials focus on safety and dosage, Phase II trials assess efficacy and side effects, and Phase III involves large-scale testing to confirm the findings and compare hydrogels with the standard treatments. Each phase demands detailed protocols and adherence to strict ethical standards [53].

Manufacturing these hydrogels under Good Manufacturing Practice (GMP) is crucial. This ensures consistent quality, sterility, and the ability to scale production. Regulatory submissions, such as the Investigational New Drug (IND) application and New Drug Application (NDA), involve extensive documentation covering clinical data and the manufacturing details. Once approved, hydrogels undergo post-market surveillance to monitor their long-term safety and effectiveness. This involves reporting adverse events, providing periodic safety updates, and being prepared for recalls if new risks are identified. Ethical considerations, particularly informed consent and patient safety, are paramount throughout this process [128].

### 6.2. Hydrogels in Hemorrhagic Stroke Treatment

Hemorrhagic stroke, characterized by the rupture of a blood vessel in the brain, poses a significant challenge due to its high mortality rate and the complexities involved in its treatment and recovery [25]. Due to the limited survival rates of patients and the technical difficulties associated with implantation and subsequent recovery processes, studies on the use of hydrogels for hemorrhagic stroke treatment remain very limited [191]. The unique challenge with hemorrhagic stroke lies in its immediate and devastating impact on the brain tissue, which is exacerbated by ongoing bleeding and the resultant pressure buildup within the skull [192]. Traditional treatments focus on controlling the bleeding and reducing intracranial pressure but often fall short in facilitating a complete recovery and preventing recurrence [193]. This is where the concept of hemostasis, or the cessation of bleeding, becomes critically important [194]. Hemostasis could potentially be a key factor in improving the outcomes for hemorrhagic stroke patients, and innovative hydrogel formulations are at the forefront of this research [195]. In their study, Barbara Verbraeken and colleagues concluded that the use of RADA16 and IEIK13 as hemostatic agents yielded histological outcomes similar to those achieved using oxidized cellulose and an inactive control. Furthermore, they demonstrated that both RADA16 and IEIK13 are effective and safe for controlling cortical bleeding in rat brains. This finding underscores the potential of these peptides as viable options for hemostasis in neurological applications, highlighting their comparable performance to existing materials while ensuring safety in a preclinical setting [196]. While this field is still in its infancy, the incorporation of hemostatic capabilities into hydrogels represents a significant advancement. These hydrogels are crafted to not only provide a temporary physical barrier to bleeding but also to support the natural clotting mechanisms of the body. By doing so, they address one of the most critical aspects of hemorrhagic stroke management: preventing further bleeding and stabilizing the patient long enough to allow for subsequent treatments and recovery processes. The development of high-performance hydrogels that can effectively manage hemostasis could be the key to overcoming the challenges that have hindered progress in this area. The current treatments are heavily impacted by the recurrent nature of bleeding and difficulty achieving a stable recovery [192]. With bleeding being a major cause of poor outcomes, the ability of these hydrogels to stop hemorrhage quickly and efficiently could revolutionize the treatment landscape. The potential of hydrogels to manage cerebral edema and hemorrhagic stroke is immense, offering innovative solutions to the long-standing challenges in stroke therapy. Table 3 summarizes the various types of hydrogels, their mechanisms of action, and their respective advantages and disadvantages for hemorrhagic stroke treatment.

## 7. The Translational Gap

Examining clinical trial registries is essential to gain a comprehensive understanding of the status of research into hydrogel-based stroke therapies. A review of the literature on hydrogel applications in stroke therapy reveals a considerable disparity between the number of preclinical studies and the extremely limited number of clinical trials on this topic. A PubMed search using the keywords “hydrogel” and “stroke” for studies from the past twenty years yields a total of 305 entries, which include 1 meta-analysis, 64 reviews, 3 systematic reviews, and 6 manuscripts related to clinical trials, though none specifically focus on hydrogel applications. Upon reviewing the National Institutes of Health’s ClinicalTrials.gov registry for hydrogel-based stroke therapy studies, as of 16 May 2024, it is notable that there are no ongoing clinical trials in this field. This stark contrast highlights the significant translational gap between preclinical studies and clinical trials. This lack of clinical trials underscores the challenges in translating promising hydrogel-based therapies from the laboratory into clinical settings. This gap indicates that despite the potential benefits observed in preclinical studies, significant barriers such as ensuring biocompatibility, optimizing the drug release mechanisms, and scaling up production must be overcome. Addressing these challenges requires a concerted effort to develop more sophisticated and representative stroke models that can reliably predict human responses. Despite these current challenges, the field of hydrogel-based stroke therapy is still relatively new and is poised to gain substantial research attention in the coming years. As the technology and our understanding of hydrogel properties continue to advance, it is anticipated that more innovative and effective hydrogel formulations will be developed.

## 8. Challenges and Future Directions

Hydrogels present an innovative and versatile platform for advancing the treatment of ischemic and hemorrhagic strokes, offering significant potential to overcome the limitations of current therapies. While preclinical studies have demonstrated their promising applications, their translation into large-scale clinical trials and widespread clinical use remains challenging. Hydrogels are advantageous in stroke therapy due to their unique properties, such as their high water content, biocompatibility, and ability to mimic the natural extracellular matrix. These characteristics facilitate their integration with host tissues and minimize adverse reactions, making them ideal candidates for delivering therapeutic agents [47]. The inherent porosity and customizable properties of hydrogels enable precise control over drug release rates, which is particularly beneficial for ischemic stroke treatment. Despite these advantages, several limitations hinder the widespread adoption of hydrogels in clinical settings. Most of the research has focused on the therapeutic effects of specific biomaterials, either alone or combined with different cell populations, often overlooking the additional cytotoxicity and inflammation the material itself may induce [124].

Typically, the compatibility of biomaterials with brain-derived neural cell populations is tested in vitro. However, in vitro studies provide a limited perspective on biomaterials’ tolerability compared to in vivo studies, which could assess how well the biomaterial integrates with the brain and the host’s biological response to grafts [137]. Many of these materials’ short- and long-term adverse effects when implanted into the brain are still unknown, as a significant challenge is the potential for immune responses. While hydrogels are generally biocompatible, certain formulations, particularly those involving synthetic polymers, could provoke immune reactions, thereby complicating their clinical use [80]. For instance, PLGA, a commonly used synthetic polymer, degrades into acidic by-products that can induce inflammation and exacerbate brain damage post-injury [137]. Notably, hyaluronic acid (HA), commonly used in experimental brain repair, does not significantly induce astrogliosis, microgliosis, or increased neuronal death in a healthy brain [201]. Certain HA hydrogels with tunable permeability have even been shown to reduce post-stroke inflammation [202]. However, some fragments of hyaluronan can activate the innate immune response, triggering alloimmunity [203]. Therefore, any biomaterial must be non-toxic in its fully polymerized form, and any by-products from its degradation must be harmless. Additionally, HA accumulation has been associated with aging and demyelinating diseases like multiple sclerosis, where HA produced by astrocytes can inhibit the remyelination of the neuronal circuits after brain damage [204,205]. While HA has shown positive effects in promoting post-stroke recovery and reducing inflammation, its usefulness in other neurological disorders remains questionable. Other biomaterials with neuro-therapeutic potential include collagen, chitosan, PLGA, and Matrigel. However, collagen poses safety concerns due to its potential contamination with viruses and prions, and it is challenging to obtain and easily degraded [206]. PLGA can degrade into by-products that exacerbate inflammation, causing additional brain damage post-injury [207]. Chitosan degrades quickly, and some preparations may cause allergic reactions [208]. Matrigel, although effective experimentally, is unsuitable for clinical applications due to its derivation from mouse sarcoma [209]. Fibrin hydrogels support cell adhesion and growth but have limitations like poor stiffness, fast degradation, and shrinkage [210]. Notably, silk fibroin emerges as a promising biomaterial for stem cell and factor therapy in brain injuries and neurodegenerative diseases. Its flexibility and adaptability have made it a staple in various biomedical applications over the years [211,212]. However, to address these challenges, several strategies are being explored. Enhancing the biocompatibility of hydrogel formulations can be achieved by developing biologically inert materials or applying surface modifications to reduce immune reactions and improve tissue integration. Optimizing the drug loading and release kinetics is also essential for therapeutic efficacy, requiring the design of hydrogel matrices with adjustable properties to control the drug release rates and durations effectively [213]. Moreover, advancements in delivery technologies, such as nanoparticle-based carriers or implantable devices, may provide more precise and targeted delivery of hydrogel-based therapies to the brain. Achieving precise control over the drug release rates and ensuring consistent degradation rates in vivo are other significant challenges.

The translational gap between preclinical research and clinical implementation is further highlighted by the limited number of clinical trials investigating hydrogel-based therapies. A review of ClinicalTrials.gov reveals only a handful of ongoing trials focused on the application of hydrogels in stroke, underscoring the need for more extensive clinical validation. This gap is evident in the contrast between the promising outcomes observed in vitro or in vivo and the lack of human trials due to the complex and variable nature of human stroke pathology. The physiological environment can vary significantly between patients, affecting hydrogel performance [11]. Additionally, the scalability of hydrogel production for clinical applications poses substantial hurdles. Large-scale manufacturing must ensure their uniformity and reproducibility, which are critical for regulatory approval and clinical success [214]. Challenges begin at the foundational level, with the current in vitro and in vivo models failing to adequately mimic the complex dynamics of human stroke pathology [215]. These models often do not replicate the full spectrum of the biological and physiological responses observed in human patients, particularly the long-term inflammatory environment and the chronic neurodegenerative processes that occur post-stroke [216].

**Figure 5:**
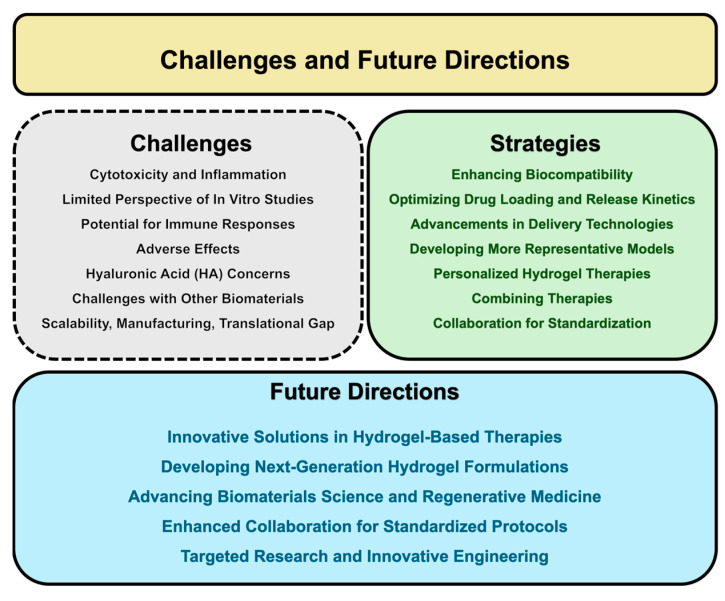
Challenges and future directions.

Looking ahead, research into hydrogel-based therapies for stroke aims to explore innovative solutions and address the current gaps in clinical practice. Personalized hydrogel therapies, tailored to the specific needs of individual patients and stroke subtypes, show promise for optimizing the treatment outcomes and reducing side effects [213]. Additionally, combining hydrogel-based therapies with other treatments, such as pharmacological interventions or rehabilitation strategies, could synergistically enhance neuroprotection and functional recovery after a stroke. Advances in biomaterials science and regenerative medicine offer exciting prospects for developing next-generation hydrogel formulations with improved properties and functionality, paving the way for groundbreaking therapies in stroke care. Addressing these challenges requires advancements in both the development of more representative human-like models of stroke and the design of hydrogels that can perform more predictably in vivo. Enhanced collaboration between researchers, clinicians, bioengineers, and regulatory bodies is also essential to establish standardized protocols that can accelerate the clinical trial process and improve the translation of hydrogel-based therapies into a clinical setup. In sum, while the potential of hydrogels in treating neurodegenerative conditions and stroke is vast, realizing this potential depends critically on closing the existing translational gaps through targeted research, innovative engineering, and rigorous testing (Figure 5).

## 9. Conclusions

This review underscores the promising potential of hydrogels in managing cerebral infarction, with applicability to both ischemic and hemorrhagic strokes. Hydrogels offer a multifaceted therapeutic approach by promoting hemostasis, enabling localized drug delivery, providing neuroprotection, supporting tissue regeneration, and mitigating oxidative stress and inflammation. Their capacity to mimic the extracellular matrix and deliver bioactive molecules directly to impacted areas could significantly enhance the effectiveness of stroke treatments while minimizing systemic side effects. The targeted delivery and sustained release of therapeutic agents from hydrogels address core challenges in stroke recovery, such as inflammation and neuronal loss, potentially reducing recurrence and promoting long-term neurological recovery. Additionally, the inclusion of growth factors can further support tissue regeneration, which is essential for restoring neurological functions and enhancing the quality of life for stroke survivors. However, despite these advantages, the translation of hydrogel-based therapies from the preclinical findings into clinical practice remains challenging. Several limitations hinder their widespread adoption, including potential immunogenic responses, the difficulty of achieving precise control over the drug release rates, and ensuring consistent degradation rates in vivo. Moreover, the scalability of hydrogel production for clinical applications poses substantial hurdles, as large-scale manufacturing must ensure their uniformity and reproducibility, which are critical for regulatory approval and clinical success. Advancing hydrogel-based therapies necessitates continued research and interdisciplinary collaboration. Large-scale trials and comprehensive evaluations are needed to confirm the efficacy and safety of these therapies across diverse patient demographics. The current discrepancy between animal models and human conditions, marked by the lack of a valid animal model for stroke and the infrequent use of aged animals, highlights the need for innovative methodologies and robust human-like models that accurately replicate stroke pathology. As the field evolves, collaborative efforts among researchers, clinicians, bioengineers, and industry partners will be essential to accelerate the development and refinement of hydrogel technologies.

## Figures and Tables

**Table 2:** Various types of hydrogels and their specific roles in the treatment of ischemic stroke, considering their unique compositions and therapeutic properties.

Hydrogel Constituent Polymers	Specific Molecules Included	Role in Ischemic Stroke
Alginate	Alginate, calcium ions	Cell delivery, tissue repair, and targeted drug release for inflammation and oxidative stress [57,95]
Collagen	Collagen, growth factors (e.g., BDNF)	Cell adhesion, promotes neurogenesis and angiogenesis, supports tissue repair [96,97]
PLGA (poly(lactic-co-glycolic acid))	PLGA, anti-inflammatory agents (e.g., dexamethasone)	Controlled release of anti-inflammatory and neuroprotective agents, supports neural regeneration [98]
Hyaluronic acid	Hyaluronic acid, extracellular matrix proteins (e.g., fibronectin)	Mimics the ECM, promotes cell proliferation and migration, delivers neuroprotective agents [99,100,101]
PEG (polyethylene glycol)	PEG, neurotrophic factors (e.g., VEGF)	Sustained release of therapeutic agents, enhances neuroprotection and tissue repair
Chitosan	Chitosan, antioxidants (e.g., Vitamin C)	Reduces oxidative stress, supports neuroprotection, and enhances tissue repair [102]
Fibrin	Fibrin, peptides (e.g., RGD peptide)	Enhances cell adhesion and tissue repair, supports neuroprotection [103]
Gelatin	Gelatin, anti-inflammatory agents	Delivers anti-inflammatory agents, supports cell proliferation and neuroprotection [104]

**Table 3:** Various types of hydrogels and their advantages and disadvantages in hemorrhagic stroke.

Hydrogel Type	Mechanism of Action	Advantages	Disadvantages
Alginate [197]	Hemostasis, supports tissue repair, delivers anti-inflammatory agents	Biocompatible, easy to gel, promotes wound healing	Potential immunogenicity, limited mechanical strength
Collagen [198]	Structural support, promotes tissue regeneration, delivers growth factors	Promotes cell adhesion, supports neurogenesis and angiogenesis	Expensive, risk of disease transmission (animal-derived)
PLGA (Poly(lactic-co-glycolic acid)) [98]	Controlled drug release, supports tissue healing	Biodegradable, tunable degradation rates, versatile	Acidic degradation products, potential inflammation
Hyaluronic Acid [191]	Mimics the extracellular matrix, reduces scarring, delivers neuroprotective agents	Hydrophilic, promotes cell proliferation and migration	Rapid degradation, requires chemical modification
PEG (Polyethylene Glycol) [191]	Reduces inflammation, supports tissue healing, delivers hemostatic agents	Non-immunogenic, easily modifiable, good biocompatibility	Non-biodegradable, potential for long-term persistence
Chitosan [199]	Structural support, reduces oxidative stress, promotes tissue regeneration	Antimicrobial, biodegradable, promotes wound healing	Variable purity, potential for allergic reactions
Fibrin [191]	Hemostasis, supports tissue regeneration, delivers therapeutic agents	Biocompatible, promotes cell adhesion and migration	Rapid degradation, risk of thrombosis
Gelatin [200]	Structural support, reduces inflammation, promotes tissue repair	Biocompatible, supports cell proliferation, inexpensive	Risk of immune response, limited mechanical strength

## Data Availability

The data presented in this study are available on request from the corresponding authors.

## Abbreviations

1. AAVs	Adeno-Associated Viruses
2. AEMA	2-aminoethyl methacrylate
3. AIBN	Azobisisobutyronitrile
4. ATRP	Atom Transfer Radical Polymerization
5. BBB	Blood–Brain Barrier
6. BDNF	Brain-Derived Neurotrophic Factor
7. CC BY	Creative Commons Attribution
8. DA	3,4-dihydroxyphenylalanine
9. ECM	Extracellular Matrix
10. EDC	3-ethylcarbodiimide hydrochloride
11. EPO	Erythropoietin
12. ESCs	Embryonic Stem Cells
13. ET	Endovascular Thrombectomy
14. FDA	Food and Drug Administration
15. FGF	Fibroblast Growth Factor
16. GDNF	Glial Cell Line-Derived Neurotrophic Factor
17. GMP	Good Manufacturing Practice
18. GMSs	Gelatin Microspheres
19. HA	Hyaluronic Acid
20. HAD	Hyaluronic Acid–Dopamine
21. HAMC	Hyaluronan/Methyl Cellulose
22. HMW	High-Molecular-Weight
23. HSCs	Hematopoietic Stem Cells
24. ICH	International Council for Harmonisation
25. IL-10	Interleukin-10
26. IND	Investigational New Drug
27. iPSC	Induced Pluripotent Stem Cell
28. IS	Ischemic Stroke
29. LAP	Lithium Phenyl-2,4,6-Trimethylbenzoylphosphinate
30. LMW	Low-Molecular-Weight
31. MAP	Microporous Annealing Particle
32. MC	Methyl Cellulose
33. MSCs	Mesenchymal Stem Cells
34. MW	Molecular Weight
35. NDA	New Drug Application
36. NGF	Nerve Growth Factor
37. NHS	N-Hydroxy Succinimide
38. NMDA	N-Methyl-D-Aspartate
39. NMP	Nitroxide-Mediated Polymerization
40. NPCs	Neural Progenitor Cells
41. NSCs	Neural Stem Cells
42. NVU	Neurovascular Unit
43. PEG	Polyethylene Glycol
44. PPi	Polyphosphate
45. PLGA	Poly(Lactic-Co-Glycolic Acid)
46. PRISMA	Preferred Reporting Items for Systematic Reviews and Meta-Analysis
47. PVA	Polyvinyl Alcohol
48. RAFT	Reversible Addition–Fragmentation Chain Transfer
49. ROS	Reactive Oxygen Species
50. SCs	Stem Cells
51. SOD	Superoxide Dismutase
52. TNF-α	Tumor Necrosis Factor-Alpha
53. tPA	Tissue Plasminogen Activator
54. VEGF	Vascular Endothelial Growth Factor
